# Decreased β-Cell Function is Associated with Cardiovascular Autonomic Neuropathy in Chinese Patients Newly Diagnosed with Type 2 Diabetes

**DOI:** 10.1007/s12264-018-0304-9

**Published:** 2018-11-15

**Authors:** Xubin Yang, Wen Xu, Yanhua Zhu, Hongrong Deng, Ying Tan, Longyi Zeng, Jianping Weng

**Affiliations:** Department of Endocrinology and Metabolism, Guangdong Provincial Key Laboratory of Diabetology, Third Affiliated Hospital of Sun Yat-sen University, Guangzhou, 510630 China

**Keywords:** Cardiovascular autonomic neuropathy, β-cell function, Type 2 diabetes mellitus

## Abstract

The influence of β-cell function on cardiovascular autonomic neuropathy (CAN), an important diabetes-related complication, is still unclear. In this study, we aimed to investigate the association between residual β-cell function and CAN in patients newly diagnosed with type 2 diabetes. We enrolled 90 newly-diagnosed type 2 diabetic patients and 37 participants with normal glucose tolerance as controls. The patients were divided into a CAN+ group (diabetic patients with CAN, *n* = 20) and a CAN− group (diabetic patients without CAN, *n = *70) according to the standard Ewing battery of tests. Fasting and postprandial plasma glucose, insulin, and C-peptide were measured. Homeostasis model assessment-beta cells (HOMA-B) and HOMA-insulin resistance (IR) were calculated. The prevalence of CAN in this population was 22.2%. Compared with the CAN− group, the CAN+ group had significantly lower fasting plasma insulin (6.60 ± 4.39 *vs* 10.45 ± 7.82 μ/L, *P = *0.029), fasting C-peptide (0.51 ± 0.20 *vs* 0.82 ± 0.51 nmol/L, *P = *0.004), and HOMA-B (21.44 ± 17.06 *vs* 44.17 ± 38.49, *P = *0.002). Fasting C-peptide was correlated with the Valsalva ratio (*r* = 0.24, *P = *0.043) and the 30:15 test (*r* = 0.26, *P = *0.023). Further analysis showed that fasting C-peptide (OR: 0.041, 95% CI 0.003–0.501, *P = *0.012) and HOMA-B (OR: 0.965, 95% CI 0.934–0.996, *P = *0.028) were independently associated with cardiovascular autonomic nerve function in this population. The patients with fasting C-peptide values < 0.67 nmol/L were more likely to have CAN than those with C-peptide levels ≥0.67 nmol/L (OR: 6.00, 95% CI 1.815–19.830, *P* = 0.003). A high prevalence of CAN was found in patients with newly-diagnosed type 2 diabetes. Decreased β-cell function was closely associated with CAN in this population.

## Introduction

Cardiovascular autonomic neuropathy (CAN), one of the most important diabetes-related complications, is closely associated with increased cardiovascular morbidity and mortality in patients with diabetes [1]. Prevention or delay of the development of CAN is clinically important [2]. However, the characteristics and risk factors of CAN are not completely clear in type 2 diabetes [1]. Although hyperglycemia is generally accepted to be the major cause of CAN [2], substantial numbers of patients with tight glycemic control still experience the progressive development of CAN, suggesting that other factors besides chronic sustained hyperglycemia play roles in the development of CAN [3]. Identification of the characteristics and the related risk factors of CAN is important to prevent or delay CAN in diabetic patients, especially at the early stage of the disease.

In recent years, both insulin and C-peptide (a peptide cleaved from proinsulin and eventually released into the bloodstream in amounts equimolar with those of insulin) have been shown to exert various influences on anti-apoptosis, metabolic diseases, and nervous system diseases [4–7]. The deterioration of β-cell function has been demonstrated to be associated with diabetic complications [8]. A previous study showed that C-peptide administration significantly improves autonomic nerve function in type 1 diabetes [9]. However, the pathophysiology of type 1 and type 2 diabetes is fundamentally different. Whether the same association of β-cell function with autonomic nerve function also occurs in patients with type 2 diabetes is poorly studied. Some studies found an association of better residual insulin secretion with decreased incidence of CAN in type 2 diabetic patients, while others did not [10–12]. Therefore, we conducted this *post hoc* analysis of our previously published multicenter, cross-sectional study to investigate the association between residual β-cell function and CAN in newly-diagnosed type 2 diabetic patients.

## Materials and Methods

### Participants

Details of the inclusion criteria and methods of this study have been published previously [13]. Briefly, the participants were enrolled from four university-affiliated hospitals in Guangdong Province, China. Ninety drug-naive patients newly diagnosed with type 2 diabetes were enrolled as the diabetes group. Exclusion criteria included uncontrolled hypertension, congestive heart failure, cardiac arrhythmias, stroke, severe liver insufficiency or renal insufficiency, proliferative retinopathy, psychiatric disease, anemia, on β-blocker or digitalis treatment within a month, pregnancy, and addiction to alcohol, cigarettes, or coffee. Forty participants with normal glucose tolerance and normal blood pressure were selected as the control group. The protocol was approved by the Ethics Committee of the Third Affiliated Hospital of Sun Yat-sen University. All participants provided written informed consent before screening.

### Measurements

All the measurements were made after a 10-h overnight fast. Waist circumference, hip circumference, and blood pressure were measured while participants were barefoot and wearing light clothes after defecation. Blood samples were collected before and 2 h after a fixed breakfast (~300 kcal) for measurement of hemoglobin A1c (HbA1c), fasting lipid profile, fasting and 2-h postprandial plasma glucose (2hPPG), insulin, and C-peptide. Fasting and postprandial plasma glucose levels, lipid concentrations for total cholesterol, triglycerides, and HDL-cholesterol (HDL-c) were measured using an automated enzymatic method (7600-020 Chemical Analyzer, Hitachi, Japan). HbA1c levels were determined by high-performance liquid chromatography (D-10 analyzer, Bio-Rad, Hercules, CA). Fasting and 2-h postprandial plasma insulin and C-peptide were measured by radioimmunoassay (Centaur XP immunoassay system, Siemens Healthcare Diagnostics, New York, NY).

Homoeostasis model assessment (HOMA) was used to estimate basal β-cell function (HOMA-B) and insulin resistance (HOMA-IR). HOMA-B was calculated as [20× fasting insulin (FINS)] / [fasting plasma glucose (FPG)—3.5]. HOMA-IR was calculated as FPG × FINS / 22.5 [14].

### Assessment of Cardiovascular Autonomic Neuropathy

Smoking and drinking coffee, wine, or tea were prohibited for at least 24 h before the tests. The tests were performed between 08:30 and 16:00 at least 2 h after breakfast in a quiet room with a temperature of 23–25°C. The standard Ewing battery of tests [15], including the Valsalva maneuver (Valsalva ratio), the deep breathing test of expiration-to-inspiration ratio (E/I test), and the lying-to-standing test (30:15 test) among the heart rate tests, and the orthostatic hypotension test, were conducted for every participant by well-trained investigators at each site who were blind to the individual’s laboratory results (details in Table 1). Sympathetic nerve dysfunction was identified by the orthostatic hypotension test, while parasympathetic nerve dysfunction was identified by the other three Ewing tests. When the autonomic nerve function was impaired, the indexes of the orthostatic hypotension test increased while the other three indexes decreased. The scores from Ewing tests were used to classify the severity of CAN (Table 2): each Ewing test was graded as normal (score = 0), borderline (score = 0.5), or abnormal (score = 1). The total score on each of the Ewing tests was calculated. The participants whose total score was ≥ 2 were considered to have CAN. Therefore, patients in the diabetes group were further divided into two sub-groups: those without CAN (CAN−) and those with CAN (CAN+). Participants with a total score ≥ 2 in the control group were excluded from further analysis to avoid any confounding impact.

**Table 1:** Sequence, body position, approximate time, and apparatus of Ewing tests.

Order	Test	Position	Approximate time (min)	Apparatus
1	Valsalva ratio	Sitting	5	ECG, modified sphygmomanometer
2	E/I	Sitting	2	ECG
3	30:15	Lying-to-standing	2	ECG
4	Orthostatic hypotension test	Lying-to-standing	3	Sphygmomanometer

E/I, deep-breathing test (expiration-to-inspiration ratio); 30:15, lying-to-standing test; ECG, electrocardiograph.

**Table 2:** Definition of normal, borderline, and abnormal in each Ewing test.

Test	Normal	Borderline	Abnormal
Valsalva ratio	≥ 1.21	1.11–1.20	≤ 1.10
E/I	≥ 15 beats/min	11–14 beats/min	≤ 10 beats/min
30:15	≥ 1.04	1.01–1.03	≤ 1.00
Orthostatic hypotension test	≤ 10 mmHg	11–29 mmHg	≥ 30 mmHg

E/I, deep-breathing test (expiration-to-inspiration ratio); 30:15, lying-to-standing test.

### Statistical Analysis

Continuous variables with a normal distribution are presented as mean ± SD. Differences between the diabetes and control groups were compared with unpaired *t*-tests. ANOVA tests with the LSD *t*-test were used to analyze differences among the CAN+, CAN−, and control groups. Pearson’s correlation coefficient was used to investigate the associations between the indexes of the Ewing tests and the indexes of residual β-cell function. The upper quartile value for fasting plasma C-peptide in the CAN+ group was 0.67 nmol/L. Accordingly, patients were separated based on the two strata of fasting plasma C peptide: patients with fasting plasma C-peptide level ≥ 0.67 nmol/L and those with fasting C-peptide < 0.67 nmol/L. Multivariate analysis was performed by binary logistic regression with CAN as the dependent variable, fasting and 2-h postprandial plasma insulin and C-peptide levels, HOMA-B, HOMA-IR and fasting plasma C-peptide strata as the independent variables to identify the factors for CAN, shown as odds ratios (OR) with 95% confidence intervals (CI). All statistical analyses were conducted with SPSS 21.0. Significance was defined as *P* < 0.05.

## Results

### Prevalence and Characteristics of Cardiovascular Autonomic Neuropathy in Patients Newly Diagnosed with Type 2 Diabetes

The results of Ewing tests in different groups are shown in Table 3. Among the diabetic patients, 22.2% (20/90) were diagnosed with CAN. From the scores of different Ewing tests, we found that the proportion of type 2 diabetic patients with abnormal sympathetic nerve function was lower than that of patients with abnormal parasympathetic nerve function. Three out of 40 participants in the control group with a total score ≥ 2 were excluded as predefined. The index of orthostatic hypotension test in the diabetes group was significantly higher than that in the control group (*P = *0.008), while the index of the Valsalva ratio test was significantly lower in the diabetes group than in the control group (*P = *0.015). All the indexes of Ewing tests differed significantly between the CAN+ group and the control group. When comparing the CAN− and control groups, only the index of the orthostatic hypotension test was different.

**Table 3:** Ewing test indexes in different groups.

	Diabetes group (*n* = 90)			Abnormality proportion of each index* n* (%)	Control group (*n* = 37)	*P* value			
	CAN+ (*n* = 20)	CAN− (*n* = 70)	Whole group			Diabetes *vs* Control	CAN+ *vs* Control	CAN− *vs* Control	CAN+ *vs.* CAN−
Valsalva ratio	1.11 ± 0.15	1.32 ± 0.26	1.27 ± 0.25	29 (32.2)	1.41 ± 0.24	0.015	<0.001	0.119	0.001
E/I(beats/min)	9.08 ± 5.76	14.47 ± 5.62	13.07 ± 6.10	30 (33.3)	15.24 ± 7.89	0.160	0.002	0.613	0.001
30:15	1.04 ± 0.07	1.15 ± 0.19	1.12 ± 0.18	9 (10)	1.19 ± 0.13	0.120	0.003	0.432	0.001
Orthostatic hypotension test (mmHg)	8.90 ± 8.67	5.17 ± 5.73	6.13 ± 6.75	1 (1.1)	2.19 ± 3.09	0.008	<0.001	0.049	0.018

CAN+, type 2 diabetic patients with cardiovascular autonomic neuropathy; CAN−, type 2 diabetic patients without cardiovascular autonomic neuropathy; E/I, deep-breathing test (expiration-to-inspiration ratio); 30:15, lying-to-standing test.

### Anthropometric and Other Laboratory Characteristics of Participants

The anthropometric and other laboratory characteristics of participants are shown in Table 4. When compared with the control group, patients in the CAN+ group were older (*P = *0.001), had higher waist circumference to hip circumference ratio (WHR) (*P = *0.015), FPG (*P* < 0.001), 2hPPG (*P* < 0.001), HBA1c (*P* < 0.001), and heart rate (*P = *0.012), while patients in the CAN− group had higher WHR (*P = *0.006), heart rate (*P = *0.013), systolic blood pressure (*P = *0.003), diastolic blood pressure (*P = *0.016), FPG (*P* < 0.001), 2hPPG (*P* < 0.001), and HBA1c (*P* < 0.001) but lower HDL-c (*P = *0.010). In the diabetes group, patients in the CAN+ group were older (*P* < 0.001) than those in the CAN− group.

**Table 4:** Anthropometric and laboratory characteristics of participants.

	Diabetes group (*n = *90)			Control group (*n = *37)	*P* value			
	CAN+ (*n = *20)	CAN− (*n = *70)	Whole group		Diabetes *vs* Control	CAN+ *vs* Control	CAN− *vs* Control	CAN+ *vs.* CAN−
BMI (kg/m^2^)	25.01 ± 4.05	25.26 ± 3.48	26.20 ± 3.59	24.56 ± 2.83	0.338	0.636	0.320	0.812
WHR	0.97 ± 0.06	0.97 ± 0.06	0.97 ± 0.06	0.93 ± 0.08	0.003	0.015	0.006	0.651
age (years)	53.70 ± 8.40	44.75 ± 9.54	46.71 ± 9.98	44.65 ± 10.74	0.301	0.001	0.961	< 0.001
Heart rate (beats/min)	79.90 ± 9.83	77.99 ± 10.12	78.42 ± 10.03	73.03 ± 8.73	0.005	0.012	0.013	0.439
SBP (mmHg)	129.40 ± 15.05	131.91 ± 16.65	131.35 ± 16.26	122.49 ± 11.89	0.003	0.103	0.003	0.516
DBP (mmHg)	78.65 ± 10.38	82.09 ± 11.69	81.31 ± 11.45	76.57 ± 10.13	0.030	0.499	0.016	0.224
TG (mmol/L)	2.27 ± 1.82	2.92 ± 2.91	2.77 ± 2.71	2.05 ± 1.37	0.141	0.741	0.080	0.294
TC (mmol/L)	5.40 ± 1.18	5.15 ± 0.85	5.21 ± 0.93	5.11 ± 0.89	0.571	0.254	0.802	0.296
HDL-c (mmol/L)	1.24 ± 0.28	1.13 ± 0.29	1.15 ± 0.29	1.29 ± 0.31	0.021	0.500	0.010	0.173
LDL-c (mmol/L)	3.07 ± 0.80	3.16 ± 0.77	3.20 ± 0.84	3.04 ± 0.81	0.323	0.222	0.461	0.457
FPG (mmol/L)	10.86 ± 2.92	9.78 ± 3.63	10.02 ± 3.49	4.86 ± 0.32	< 0.001	< 0.001	< 0.001	0.146
2hPPG (mmol/L)	17.73 ± 5.19	15.46 ± 5.14	15.91 ± 5.19	5.85 ± 1.05	< 0.001	< 0.001	< 0.001	0.077
HBA1c (%)	9.53 ± 2.14	9.21 ± 2.06	9.28 ± 2.07	5.38 ± 0.47	< 0.001	< 0.001	< 0.001	0.474

CAN+, type 2 diabetic patients with cardiovascular autonomic neuropathy; CAN−, type 2 diabetic patients without cardiovascular autonomic neuropathy; BMI, body mass index; WHR, waist-hip ratio; SBP, systolic blood pressure; DBP, diastolic blood pressure; TG, triglyceride; TC, total cholesterol; HDL-c, high-density lipoprotein cholesterol; LDL-c, low-density lipoprotein cholesterol; FBG, fasting blood glucose; 2hPPG, 2-h postprandial plasma glucose; HBA1c, hemoglobin A1c.

### Comparison of β-Cell Function and Insulin Resistance Among Groups

**Fig. 1:**
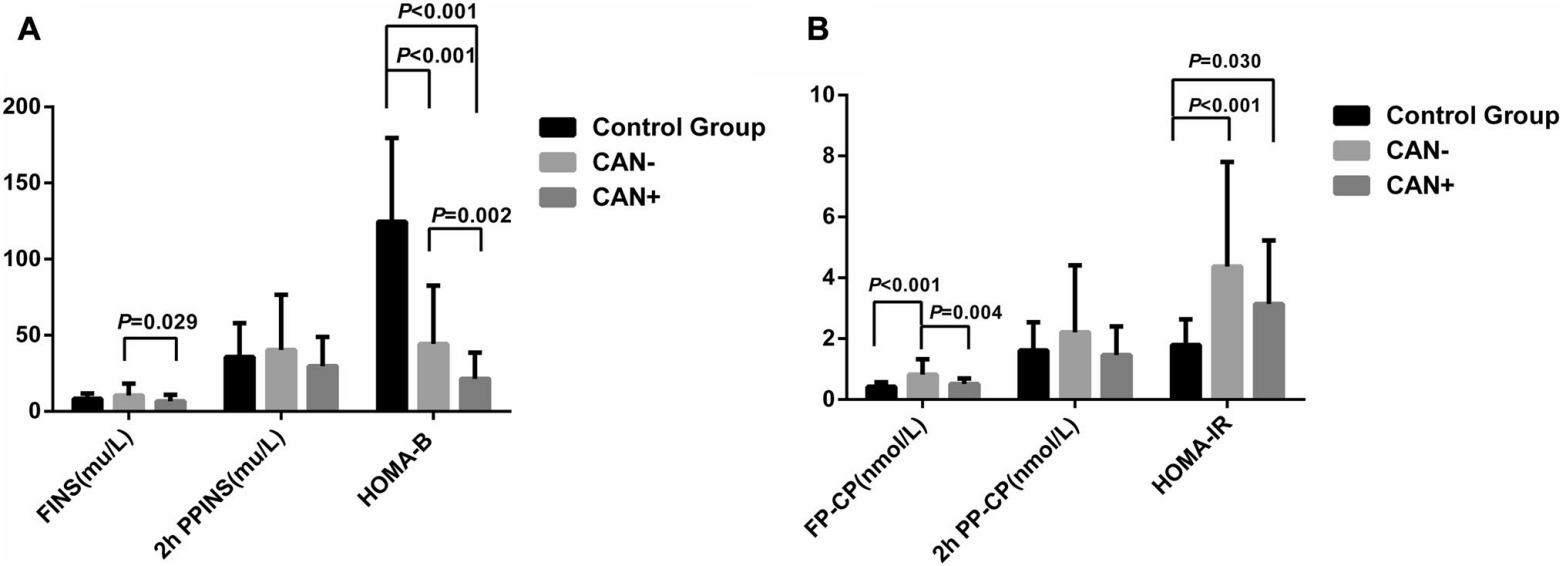
Comparison of β-cell function indexes among groups. Comparisons of FINS, 2h PPINS and HOMA-B (**A**) and FP-CP, 2h PP-CP, and HOMA-IR (**B**) among the CAN+, CAN−, and control groups. FINS, fasting plasma insulin; 2h PPINS, 2-h postprandial plasma insulin; FP-CP, fasting plasma C-peptide; 2h PP-CP, 2-h postprandial plasma C-peptide; CAN+, type 2 diabetic patients with cardiovascular autonomic neuropathy; CAN−,type 2 diabetic patients without cardiovascular autonomic neuropathy.

Compared with the control group, patients in the CAN+ group had lower HOMA-B (21.44 ± 17.06 *vs* 124.58 ± 55.04, *P* < 0.001) but higher HOMA-IR (3.14 ± 2.09 *vs* 1.80 ± 0.84, *P = *0.030); and the CAN− group had lower HOMA-B (44.17 ± 38.49 *vs* 124.58 ± 55.04, *P* < 0.001) but higher fasting plasma C-peptide (0.82 ± 0.51 *vs* 0.42 ± 0.15 nmol/L, *P* < 0.001) and HOMA-IR (4.36 ± 3.43 *vs* 1.80 ± 0.84, *P* < 0.001) (Fig. 1). In the diabetes group, FINS, fasting plasma C-peptide, and HOMA-B in the CAN+ group were significantly lower than those in the CAN− group (FINS: 6.60 ± 4.39 *vs* 10.45 ± 7.82 mu/L, *P = *0.029; fasting plasma C-peptide 0.51 ± 0.20 *vs* 0.82 ± 0.51 nmol/L, *P = *0.004; and HOMA-B 21.44 ± 17.06 *vs* 44.17 ± 38.49, *P = *0.002). There was no significant difference in HOMA-IR between the CAN+ and CAN− groups.

### Pearson’s Correlation Between Ewing Test Indexes and Residual β-Cell Function

Pearson’s correlation between the Ewing test indexes and residual β-cell function indexes (Table 5) showed that fasting C-peptide was positively correlated with Valsalva ratio in the CAN+ group (*r = *0.48, *P = *0.046), the CAN− group (*r = *0.37, *P = *0.005), and the entire diabetes group (*r = *0.24, *P = *0.043). Fasting C-peptide was also correlated with the 30:15 test in the CAN− group (*r = *0.34, *P = *0.016) and the entire diabetes group (*r = *0.26, *P = *0.023). However, no significant relationship of the Ewing test and residual β-cell function indexes was found in the control group.

**Table 5:** Pearson’s correlation between Ewing tests and residual β-cell function indexes in diabetes groups.

	Fasting plasma insulin						2h postprandial plasma insulin					
	CAN+		CAN−		Entire group		CAN+		CAN−		Entire group	
	*r*	*P*	*r*	*P*	*r*	*P*	*r*	*P*	*r*	*P*	*r*	*P*
Valsalva ratio	0.32	0.211	0.20	0.140	0.12	0.318	0.28	0.269	0.25	0.071	0.20	0.103
E/I	0.27	0.291	0.16	0.246	0.01	0.991	0.12	0.659	0.11	0.448	0.02	0.893
30 :15 test	0.42	0.095	0.06	0.694	0.13	0.297	0.23	0.374	0.12	0.396	0.02	0.848
Orthostatic hypotension test	0.19	0.462	−0.03	0.831	−0.05	0.678	−0.02	0.944	−0.11	0.443	−0.06	0.648

	Fasting plasma C-peptide						2-h postprandial plasma C-peptide					
	CAN+		CAN−		Entire group		CAN+		CAN−		Entire group	
	*r*	*P*	*r*	*P*	*r*	*P*	*r*	*P*	*r*	*P*	*r*	*P*
Valsalva ratio	0.48	0.046*	0.37	0.005*	0.24	0.043*	0.29	0.257	0.02	0.901	0.05	0.674
E/I	0.17	0.512	0.02	0.914	0.12	0.318	0.07	0.784	0.10	0.458	0.03	0.810
30 :15 test	0.17	0.500	0.34	0.016*	0.26	0.023*	0.03	0.990	0.01	0.918	0.15	0.200
Orthostatic hypotension test	−0.10	0.685	−0.13	0.341	−0.01	0.915	−0.24	0.350	−0.02	0.908	−0.08	0.515

	HOMA-IR						HOMA-B					
	CAN+		CAN−		Entire group		CAN+		CAN−		Entire group	
	*r*	*P*	*r*	*P*	*r*	*P*	*r*	*P*	*r*	*P*	*r*	*P*
Valsalva ratio	0.32	0.215	0.20	0.148	0.13	0.259	0.19	0.457	0.16	0.255	0.17	0.154
E/I	0.15	0.558	0.13	0.347	0.04	0.754	0.34	0.183	0.16	0.249	0.12	0.313
30 :15 test	0.35	0.173	0.13	0.368	0.13	0.265	0.38	0.136	0.08	0.558	0.06	0.649
Orthostatic hypotension test	−0.19	0.459	−0.08	0.591	−0.08	0.511	−0.06	0.829	−0.06	0.665	−0.07	0.574

CAN+, type 2 diabetic patients with cardiovascular autonomic neuropathy; CAN−, type 2 diabetic patients without cardiovascular autonomic neuropathy; E/I, deep-breathing test (expiration-to-inspiration ratio); 30:15 test, lying-to-standing test; *P < 0.05.

### Logistic Regression Analysis of the Presence of CAN and Residual β-Cell Function Indexes

Logistic regression was calculated to evaluate the independent association of the presence of CAN with fasting and 2-h postprandial plasma insulin and C-peptide levels, HOMA-B, HOMA-IR, and fasting plasma C-peptide strata.

**Fig. 2:**
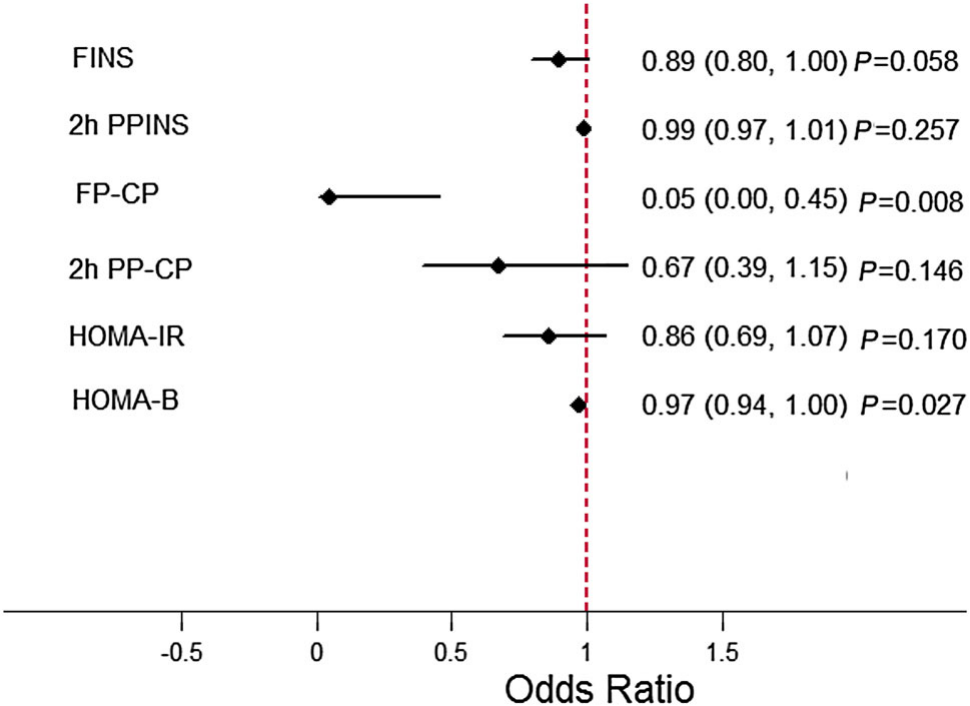
Multivariate analysis of residual β-cell function indexes and the presence of CAN. FINS, fasting plasma insulin; 2h PPINS, 2-h postprandial plasma insulin; FP-CP, fasting plasma C-peptide; 2h PP-CP, 2-h postprandial plasma C-peptide; CAN+, type 2 diabetic patients with cardiovascular autonomic neuropathy; CAN−, type 2 diabetic patients without cardiovascular autonomic neuropathy.

Fasting plasma C-peptide and HOMA-B were independently and significantly associated with the presence of CAN (*P = *0.008 and *P = *0.027, respectively). No association of FINS (*P = *0.058), 2-h postprandial plasma insulin (*P = *0.257), 2-h postprandial plasma C-peptide (*P = *0.146), and HOMA-IR (*P = *0.170) with the presence of CAN was found (Fig. 2). After adjusting for age, fasting plasma C-peptide and HOMA-B were still significantly associated with the presence of CAN (OR, 0.041; 95% CI, 0.003–0.501; *P = *0.012 and OR, 0.965; 95% CI, 0.934–0.996; *P = *0.028, respectively).

**Fig. 3:**
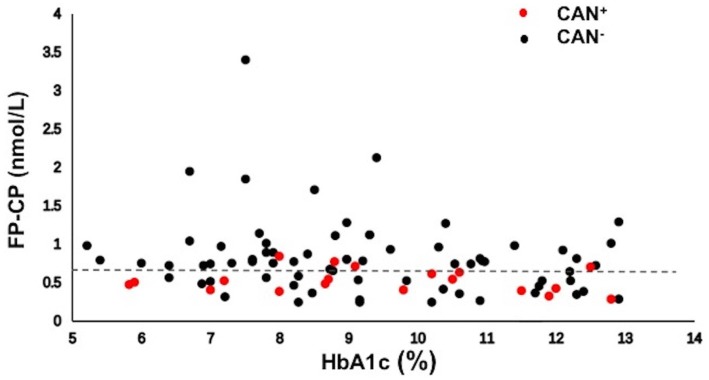
Association between C-peptide strata and CAN. FP-CP, fasting plasma C-peptide; HbA1c, hemoglobin A1c; CAN, cardiovascular autonomic neuropathy; CAN+, type 2 diabetic patients with cardiovascular autonomic neuropathy; CAN−, type 2 diabetic patients without cardiovascular autonomic neuropathy.

There were 20 patients with CAN and 70 without CAN in the diabetes group (Fig. 3). Importantly, when the data were plotted against HbA1c, an independent association was found between low levels of fasting plasma C-peptide and CAN+. More specifically, a fasting plasma C-peptide level ≥0.67 nmol/L, regardless of HbA1c, was associated with less CAN+. And the patients with C-peptide values <0.67 nmol/L were 6 times more likely to have CAN than those with C-peptide levels ≥0.67 nmol/L (OR, 6.00; 95% CI, 1.815–19.830; *P = *0.003) (Fig. 3).

## Discussion

CAN, the possible cause of asymptomatic ischemia or myocardial infarction (MI), has been found to be associated with increased mortality in patients with diabetes, yet it remains one of the most overlooked complications of diabetes [16]. Previous studies have demonstrated that CAN is found in patients with newly-diagnosed diabetes, pre-diabetes, and even the first-degree relatives of type 2 diabetic patients with normal glucose tolerance [17, 18]. Although CAN progresses with the duration of diabetes [19], it can be reversed at the very early stage by interventions on risk factors [20], just like other nervous system diseases [21]. Hence, it is of great clinical importance to identify CAN and its characteristics in the early stage of the disease.

In this study, we targeted on newly-diagnosed and drug-naïve type 2 diabetic patients for the early detection of CAN using the standard Ewing battery of tests. Our results showed that the index of the orthostatic hypotension test in the diabetes group was higher than that in the control group, while the index of the Valsalva ratio test was significantly lower in the diabetes group. These findings revealed an autonomic dysfunction in newly-diagnosed type 2 diabetic patients. Moreover, our results demonstrated that parasympathetic nerve activity was more commonly and severely impaired than sympathetic nerve activity in this population. Eugenia Rota [22] found that the E/I test is abnormal in 28% of newly-diagnosed type 2 diabetic patients, and postural hypotension is present in 6% of that population, similar to our findings. However, sympathetic dysfunction (51.9%) has been found to be slightly more common than parasympathetic dysfunction (44.2%) in an Indian population with a relatively longer duration of diabetes [23]. These results might further suggest that parasympathetic nerve function is more vulnerable to hyperglycemia than sympathetic nerve function. Therefore, CAN, especially parasympathetic nerve dysfunction, can be detected once type 2 diabetes is diagnosed.

Hyperglycemia is a crucial pathophysiological factor in the development of CAN [2]. Previous studies have shown that autonomic nerve function improves significantly after blood glucose is well-controlled [1]. However, the fact that even achieving tight glycemic control may not prevent the progression of CAN [8] implies the importance of better understanding and exploring other related factors. In recent years, the role of β-cell function in diabetes-related complications has been demonstrated in different studies. Studies of type 1 diabetes suggest that residual endogenous insulin secretion reflected by C-peptide concentrations may have favorable effects on protection against the microvascular complications. In the Diabetes Control and Complications Trial, higher and sustained levels of stimulated C-peptide are associated with reduced incidence of retinopathy, nephropathy, and hypoglycemia [24]. In another study, urinary albumin excretion along with heart rate variability significantly improved in type 1 diabetic patients who received 6-month combination treatment of C-peptide and insulin [9], indicating that C-peptide and insulin improve renal and autonomic nerve function in type 1 diabetes. The potential mechanisms for these effects include the roles of C-peptide in the activation of Na/K channels, anti-inflammatory action, and improvement of nitric oxide bioavailability and endothelial function [25]. Type 2 diabetes has an underlying pathophysiology completely different from type 1 diabetes, especially the β-cell functional deterioration at the onset and development of the disease. And the findings on the relationship between β-cell function and diabetic complications in patients with type 2 diabetes are still controversial. Studies have shown that reduced β-cell function, reflected by lower fasting C-peptide and stimulated C-peptide levels, is associated with diabetic retinopathy, nephropathy, and peripheral neuropathy but not macrovascular complications in type 2 diabetic patients [26]. However, another study found an association between serum C-peptide and macrovascular rather than microvascular diabetic complications [27]. Nevertheless, little attention has been paid to the association between residual β-cell function and CAN in type 2 diabetes. In the present study, we found that diabetic patients with CAN were characterized by decreased residual β-cell function compared with diabetic patients without CAN and the control group, represented by lower fasting plasma insulin, plasma C-peptide, and HOMA-B. Moreover, further analysis revealed that the fasting plasma C-peptide and HOMA-B were independently and closely associated with CAN. More interestingly, our data showed that a fasting plasma C-peptide level <0.67 nmol/L was associated with the presence of CAN in this population, regardless of HbA1c levels. Taken together, our results support a close relationship of β-cell function with the presence of CAN in newly-diagnosed type 2 diabetic patients. We note that some other studies have contrary results. A previous study found that a higher serum C-peptide level is significantly associated with the presence of autonomic neuropathy [27]. We considered several reasons for this discrepancy, including the different study designs, ethnicity, degree of obesity, the longer duration of diabetes, and adoption of the orthostatic test but not the standard Ewing battery of tests to assess CAN in that study. With the longer disease duration and the impact of various hypoglycemic agents used by the patients, the residual β-cell function of the patients in that study differed to a great extent from the newly-diagnosed and drug-naïve patients in our study. Regarding the method used to assess CAN, the orthostatic test was used to identify sympathetic nerve dysfunction but not all autonomic function. Indeed, these differences among the studies again highlight the complexity of the relationship between β-cell function and CAN in type 2 diabetes.

One of the strengths of this study is that the participants were newly-diagnosed and drug-naïve patients, so that many confounding factors like disease duration and impact of hypoglycemic agents were avoided. By revealing the relationship between the residual β-cell function and CAN in this population, our results suggested that in the attempt to delay the natural course of CAN, preserving β-cell function should be one of the important strategies of treating type 2 diabetes once it is diagnosed. Certain strategies, such as initiating insulin therapy as early as possible and avoiding drugs that over-stimulate β-cell function might be advocated to rescue β-cell function and eventually protect autonomic function. Another strength of our study is the adoption of the standard Ewing battery of tests to guarantee validation of the CAN diagnosis.

Some limitations of this study should be addressed in the interpretation of the results. The sample size was relatively small. And as the patients in this study were enrolled under hospital-based circumstances, they might not be the representative of the entire population of diabetic patients. And it might seem to be a limitation that other methods to detect CAN such as heart rate variability (HRV) or heart rate turbulence (HRT) [28, 29] were not used in our study. According to the Toronto Diabetic Neuropathy Expert Group, cardiovascular reflex tests (Ewing tests) are recommended as the gold standard in clinical autonomic testing because of the good sensitivity, specificity, and reproducibility, as well as being noninvasive, safe, well standardized, and easily performed [1]. Besides, HRV and HRT have no definite standard values for the diagnosis of CAN as the Ewing tests do [28]. That is why we decided to use Ewing tests. Therefore, we consider that our findings provide suggestive hints of an independent and close relationship of residual β-cell function with CAN in newly-diagnosed type 2 diabetic patients. The protection of β-cell function should not be neglected as an important component of the management of diabetes when therapeutic strategies are considered.

## Conclusions

Cardiovascular autonomic nerves have already been damaged when type 2 diabetes mellitus is diagnosed. Parasympathetic nerves are more commonly and severely impaired than sympathetic at the early stage of the disease. Decreased β-cell function is associated with the presence of CAN in this population. Better residual β-cell function might be associated with decreased incidence of CAN in newly-diagnosed patients with type 2 diabetes.
